# MicroRNA profile in the squamous cell carcinoma: prognostic and diagnostic roles

**DOI:** 10.1016/j.heliyon.2020.e05436

**Published:** 2020-11-06

**Authors:** Soudeh Ghafouri-Fard, Mahdi Gholipour, Mohammad Taheri, Zeinab Shirvani Farsani

**Affiliations:** a Department of Medical Genetics, Shahid Beheshti University of Medical Sciences, Tehran, Iran; b Urogenital Stem Cell Research Center, Shahid Beheshti University of Medical Sciences, Tehran, Iran; c Department of Cell and Molecular Biology, Faculty of Life Sciences and Technology, Shahid Beheshti University G.C., Tehran, Iran

**Keywords:** Bioinformatics, Cell biology, Cancer research, Epigenetics, Genetics, Gene expression, Gene regulation, Genomics, Squamous cell carcinoma, miRNA, Biomarker, Expression, Cancer

## Abstract

Head and neck squamous cell carcinomas (HNSCCs) are human malignancies associated with both genetic and environmental factors. MicroRNAs (miRNAs) as a group of small non-coding RNAs have prominent roles in the development of this kind of cancer. Expressions of several miRNAs have been demonstrated to be increased in HNSCC samples vs. non-malignant tissues. *In silico* prediction tools and functional analyses have confirmed the function of some miRNAs in the modulation of cancer-associated targets, thus indicating these miRNAs as onco-miRs. Moreover, numerous miRNAs have been down-regulated in HNSCC samples. Their targets mostly enhance cell proliferation or inhibit apoptosis. miRNAs signature has practical implications in the diagnosis, staging, and management of HNSC. Most notably, numerous miRNAs have been shown to alter response of tumor cells to anti-cancer drugs such as cisplatin and doxorubicin. Circulating levels of these small transcripts have been suggested as promising biomarkers for diagnosis of HNSCC. In the present manuscript, we sum up the available literature regarding the miRNAs signature in HNSCC and their role as diagnostic/prognostic biomarkers.

## 1 Introduction

Squamous cell carcinoma has been detected in various regions in the head and neck. These cancers have several risk factors including tobacco and alcohol usage for tumors of the oral cavity, oropharynx, hypopharynx, and larynx. Moreover, oncogenic viruses are among the most important risk factors for cancers of the nasopharynx, palatine, and lingual tonsils. Based on the rapid increase in the frequency of human papillomavirus (HPV)–associated oropharyngeal cancer, the incidence of these cancers are expected to exceed the incidence of cervical cancer [1]. The presence of distant metastasis at the time of diagnosis and high occurrence of inoperable local and regional relapses following the primary therapeutic modalities are associated with high mortality rate of HNSCC [2]. Mutations in tumor suppressor genes such as *TP53* are detected in head and neck squamous carcinomas (HNSCCs) triggered by smoking and alcohol consumption. Yet, the HPV-positive HNSCCs have their specific expression signature and DNA methylation profiles [3]. In addition to the recurrent mutations in the tumor suppressor genes and differentiation pathways [1], HNSCCs are associated with dysregulation of several microRNAs (miRNAs) [4]. These transcripts are initially produced as primary miRNAs which are afterward processed into pre-miRNA hairpin configurations. Subsequently, these hairpins are processed into short double strand RNA (dsRNA) structures. Ultimately, one strand of this dsRNA produces the mature miRNA [5]. This endogenous small transcripts control expression of their targets at the post–transcriptional level through binding with the 3′ UTR of the mRNA [6]. Based on the significance of miRNAs in the modulation of cell proliferation, differentiation and apoptosis, these small RNAs influence carcinogenesis process and therefore are putative biomarkers in this regard [7]. In the present paper, we summarize the current literature on signature and function of miRNAs in HNSCC. We investigated the PubMed/Medline and google scholar databases with the key words “micoRNA” or “miRNA” AND “head and neck squamous cell carcinoma” to retrieve related articles published until February 2020. We firstly assessed the abstract to verify the relevance of articles with the topic of the narrative review. Then, two authors independently went through the main text and extracted the data regarding assessed samples (numbers and characteristics), details of in vitro experiments (cell lines, identified targets and related signaling pathway, functional importance of the miRNA) and association between the dysregulated miRNAs and clinical outcome. Subsequently, we summarized the obtained data in Tables.

## 2 Onco-miRNAs in HNSCC

**Figure 1:**
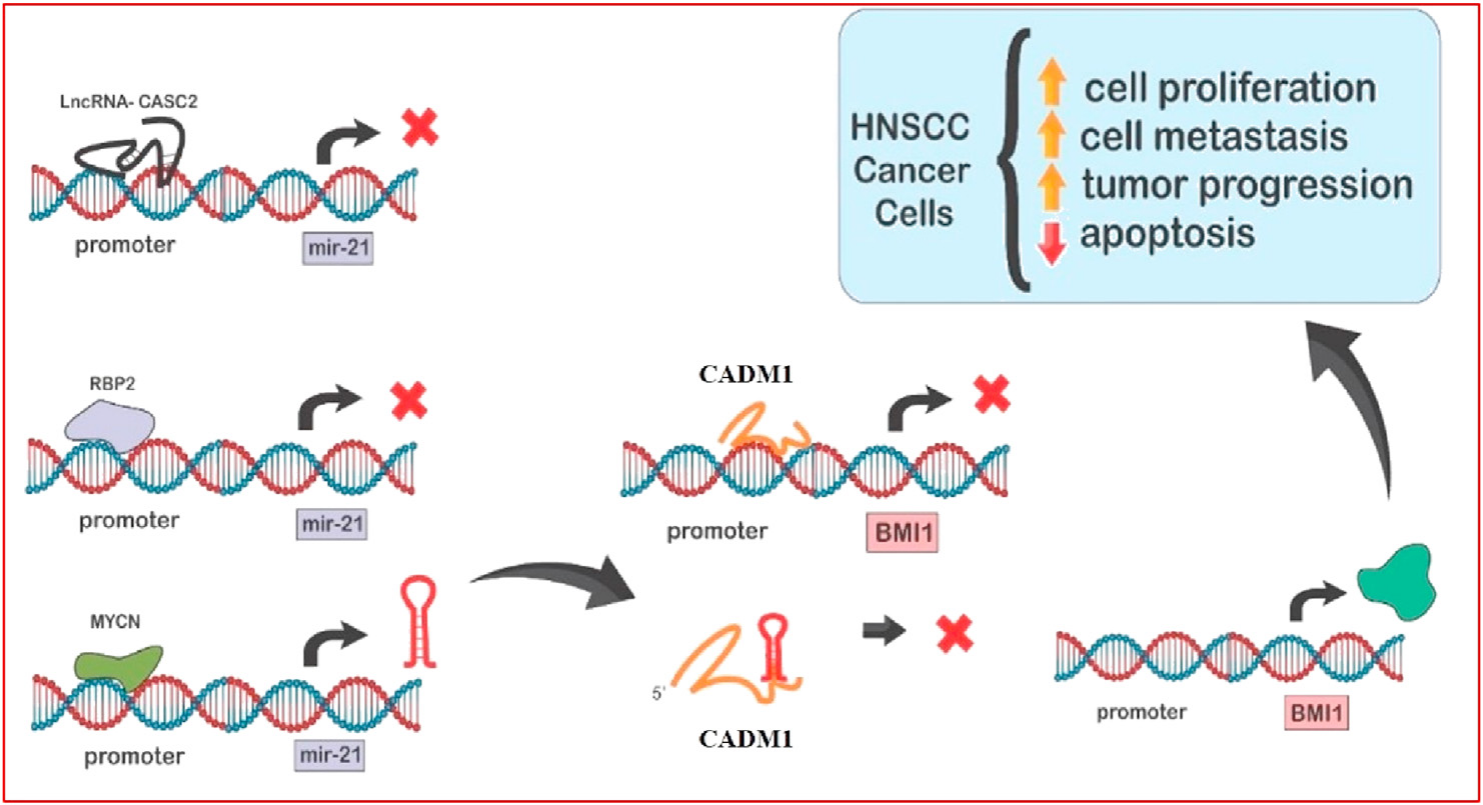
Molecular mechanism of participation of miR-21 in HNSCC. Upregulation of the histone demethylase RBP2 and long non-coding RNA CASC2 inhibit miR-21 expression, while MYCN directly interacts with the promoter of miR-21 and increases miR-21 expression. miR-21 suppresses CADM1 expression, consequently increases chemo-resistance in HNSCC. Downregulation of the tumor suppressor gene CADM1 by miR-21 results in enhanced BMI1 expression, which in turn enhances tumor progression, proliferation, metastasis, and reduction of apoptosis in HNSCC cells [10].

We extracted the data of up-regulated miRNAs in HNSCC tumors compared with non-malignant tissues and constructed a Table. Totally, we included information of 63 articles showing up-regulation of miRNAs in this kind of cancer in Table 1. *In silico* prediction tools and functional analyses have confirmed the function of some of these miRNAs in the regulation of cancer-associated targets, thus indicating these miRNAs as onco-miRs. For instance, Ramdas et al. have measured expression of miRNAs in HNSCC and their corresponding normal tissues using miRNA bioarrays. They showed differential expression of 20 miRNAs between these specimens. Authors have also shown down-regulation of targets of these miRNAs. Among these targets were adenomatous polyposis coli (APC), programmed cell death protein 4 (PDCD4) and TGF beta receptor 3 (TGFBR3), thus concluding that over-expression of these miRNAs might contribute in the down-regulation of mRNAs that control growth and progression of HNSCC [8]. Kalfert et al. have reported high levels of miR-21, miR-200c and miR-34a in HNSCC tumors of all assessed sites. Notably, expression levels of miR-34a in tumors were correlated with p16 expression [9]. Figure 1 shows underlying mechanism for participation of miR-21 in this kind of cancer.

**Table 1:** List of up-regulated miRNAs in HNSCC.

Author/year	microRNA	Cancer subtype	Tissues	Clinical samples	Cell line	Targets/Regulators	Signaling Pathways	Function	Clinical outcome	Ref
Kalfert et al., 2015	miR-200c, miR-34a, miR-21	HNSCC (oropharyngeal, laryngeal and hypopharyngeal carcinomas)	Tumor and normal tissues	51 HNSCC patients	-	-	-	Have some potential prognostic significance	-	[9]
Ganci et al., 2017	miR-429, miR96-5p, miR-21-5p, miR-21-3p	HNSCC	Tumor and normal tissues	132 HNSCC patients	Cal27 line	CHK2 and EZH2	cell cycle pathway	These miRNAs are predictors of recurrence when highly expressed.	-	[11]
Childs et al., 2009	miR-21	HNSCC	Tumor and normal tissues	104 HNSCC patients	-	PDCD4, ACTA2, BTG-2		miR-21 via inhibition of PDCD4, ACTA2, and BTG-2 could promote invasion and metastasis.		[12]
Hebert et al., 2007	miR-98	HNSCC	-	-	SCC-4, SCC-9, UMB-10B	HMGI-C	-	miR-98 diminished mRNA expression of HMGA2 and increased cell survival during hypoxia.	-	[13]
Lubov et al., 2017	miR-7, miR-9, miR-15, miR-18, miR-19, miR-21, miR-23, miR-24, miR-93, miR-96, miR-99, miR-130, miR-139, miR-141, miR-155, miR-181, miR-195, miR-196, miR-210, miR-211, miR-214, miR-222, miR-296, miR-302, miR-331, miR-345, and miR-424	HNSCC	Tumor and normal tissues	A meta-analysis study includes 8,194 subjects with HNSCC	-	-	apoptotic and cell death signaling pathways	-	Significant elevated expressions of these miRNAs were associated with poor prognosis in HNSCC.	[14]
Hou et al., 2015	miR-223	HNSCC	Tumor and normal tissues And plasma samples of patients prior and 6 months after surgical removal of tumor	16 HNSCC patients and 9 paired plasma samples	-	FBXW7/hCdc4	FGF cell signaling	Dysregulation of plasma miR-223 is a biomarker for cancer recurrence.	-	[15]
Summerer et al., 2015	miR-142-3p, miR-186-5p, miR-195-5p, miR-374b-5p and miR-574-3p	HNSCC (oropharyngeal, laryngeal and hypopharyngeal carcinomas)	Tumor and normal tissues And Plasma	18 HNSCC patients and 12 healthy donors, tumor biopsies of 10 out of the 18 patients	-	-	-	These miRNAs are HPV-independent markers for HNSCC prognosis in persons treated with combined radiochemotherapy.	Up-regulation of miR-186-5p, miR-374b-5p and miR-574-3p before treatment correlated with reduced PFS and/or OS. Up-regulation of miR-28-3p, miR-142-3p, miR-191-5p, miR-195-5p, miR-425-5p and miR-574-3p after treatment was correlated with poor prognosis.	[16]
GOMBOS et al., 2013	miR-21, miR-155, miR-191	OSCC	Tumor and normal tissues	40 HNSCC patients	-			These oncomirs are promising genomic biomarkers for early-cancer detection.		[17]
GOMBOS et al., 2013	miR-221	OSCC	Tumor and normal tissues	40 HNSCC patients	-	*PTEN, TIMP3*	AKT pathway	miR-221 increases *TRAIL* resistance and promotes cellular migration via activation of the AKT pathway and metallopeptidases.		[17]
Chen et al., 2019	miRNA-10a	OSCC	Tumor and normal tissues	52 HNSCC patients	SCC090,SCC25	GLUT1	-	miRNA-10a up-regulation enhances glucose uptake and cell proliferation.	-	[18]
Schneider et al., 2018	hsa-miR-32-5p	OSCC	Tumor and normal tissues And serum	5 HNSCC patients	-	-	-	Marker for OSCC detection	-	[19]
Schneider et al., 2018	hsa-miR-21-5p	OSCC	Tumor and normal tissues And serum	5 HNSCC patients	-	PTEN	PI3k/Akt pathway and rapid cell growth	Marker of survival and response to treatment	-	[19]
Schneider et al., 2018	hsa-miR-375	OSCC	Tumor and normal tissues And serum	5 HNSCC patients	-	MMP13	-	Increases metastatic potential and aggressiveness	-	[19]
Schneider et al., 2018	hsa-miR-31-3p	OSCC	Tumor and normal tissues And serum	5 HNSCC patients	-	Nanog/OCT4/Sox2/EpCAM	-	hsa-miR-31 is an important regulator of tumor suppressor genes, and associated with decreased survival, and increased cell proliferation.	-	[19]
Manikandan et al., 2016	miR-144	OSCC	Tumor and normal tissues	discovery cohort (n = 29), validation cohort (n = 61), 9 independent normal oral specimens	-	PTEN	PI3K/Akt signaling pathway	Associated with regional lymph node invasion	-	[20]
Liu et al., 2012	miR-31	OSCC	Salivary	45 patients with OSCC and 24 controls	SAS	HIF	hypoxia pathways	Biomarker for early detection and postoperative follow-up	-	[21]
Lu et al., 2016	miR-31	HNSCC	Tumor and normal tissues	58 HNSCC patients	SAS, OECM1, FaDu and HSC3 HNSCC cells, 293T cell	ARID1A	Nanog/OCT4/Sox2/EpCAM	miR-31 suppresses ARID1A and enhances the oncogenicity and stemness of HNSCC	-	[22]
Rock et al., 2019	HNnov-miR-3	OSCC	Tumor and normal tissues	25 tumor and 5 non-malignant tissue samples	-	-	-	Prognostic marker for recurrence-free and overall survival	-	[23]
Rock et al., 2019	HNnov-miR-2, HNnov-miR-30	OSCC	Tumor and normal tissues	25 tumor and 5 non-malignant tissue samples	-	-	-	significantly associated with HPV status	-	[23]
Salazar-Ruales et al., 2018	miR-122-5p	HNSCC	saliva samples	108 HNSCC patients and 108 controls	-	-	-	a specific biomarker for the diagnosis of HNSCC	-	[24]
Salazar-Ruales et al., 2018	miR-146a-5p	HNSCC	saliva samples	108 HNSCC patients and 108 controls	-	kinase-1 associated with the interleukin-1 receptor	NF-κB pathway	Inhibits the expression of the kinase-1 associated with the interleukin-1 receptor, participates in the NF-κB pathway	-	[24]
Schneider et al., 2018	miR-21-5p	OSCC	Tumor and normal tissues and serum	five patients	-	PTEN	Pi3k/Akt pathway	Regulates cell growth and proliferation by targeting PTEN, biomarker for survival and response to treatment	-	[19]
Schneider et al., 2018	miR-375	OSCC	Tumor and normal tissues and serum	five patients	-	MMP13	-	Predictor of prognosis	-	[19]
Schneider et al., 2018	hsa-miR-32-5p	OSCC	Tumor and normal tissues and serum	five patients	-	-	-	Marker for non-invasive diagnosis of patients with OSCC	-	[19]
Hu et al., 2015	miR-223, miR-142-3p, miR-16, miR-23a	laryngeal SCC	Tumor and normal tissues	46 patients	-	-	-	Monitoring biomarkers for laryngeal SCC	-	[25]
Hu et al., 2015	miR-21	laryngeal SCC	Tumor and normal tissues	46 patients	-	PDCD4	-	Suppresses tumor growth through decreasing the tumor suppressor tropomyosin I	-	[25]
Victoria Martinez et al., 2015	miR-103/miR-107	HNSCC (oral, oropharyngeal, laryngeal)	Serum	7 males with HNSCC and 7 healthy control males	-	DAPK, KLF4, and NF1	-	An oncomiR that promotes cell proliferation and migration	-	[26]
Victoria Martinez et al., 2015	miR-320	HNSCC (oral, oropharyngeal, laryngeal)	Serum	7 males with HNSCC and 7 healthy control males	-	CDKN2A and PTEN	-	Promotes proliferation by suppression of the cell cycle inhibitors p57 and p21	-	[26]
Ries et al., 2017	miR-3651 and miR-494	OSCC	Whole blood	21 patients with recurrence of OSCC and 21 patients without recurrence	-	-	-	Prognostic factor, useful in design of a minimally invasive strategy for the detection and monitoring of OSCC	-	[27]
Hung et al., 2016	miR-21 and miR-31	OPMD	Tumor and normal tissues and saliva	20 saliva samples and 46 tissue samples from patients with OPMD	-	-	-	Salivary miR-21 and miR-31 are useful for cancer screening. Epithelial dysplasia and miR-31 up-regulation are markers for recurrence and/or malignant transformation.	-	[28]
RIES et al., 2014	miR-3651 and miR-494	OSCC	Whole blood	50 patients and 33 healthy controls	-	-	-	Suppress cell-cycle arrest, cell senescence, and apoptosis	-	[29]
Xiao et al., 2015	miR-93	Laryngeal SCC	Tumor and normal tissues	59 patients	HEK293 and Hep-2	cyclin G2	CCNG2-MMP-9 pathway	Enhanced cell proliferation, reduced apoptosis rates, induced cell cycle arrest and enhanced cell migration and invasion	-	[30]
Geng et al., 2016	miR-365a-3p	Laryngeal SCC	-	-	Human Hep-2	p-AKT (Ser473)	PI3K/AKT signaling pathway	Promotes cell cycle progression, migration, invasion, tumor growth and metastasis, and suppresses cell apoptosis in laryngeal squamous cell carcinoma	-	[31]
Xu et al., 2016a	miR-483-5p	OSCC	sera samples	101 OSCC patients	-	-	-	Prognostic factor	High serum miR-483-5p expression predicted poor overall survival. Elevated miR-483-5p was predictive for nodal metastases, late cancer stages, and poor prognosis.	[32]
Baba et al., 2016	miR-155-5p	OSCC	Tumor and normal tissues	73 patients	HaCaT and HSC-3	SOCS1	STAT3 signaling pathway	miR-155-5p enhanced OSCC-cell migration rather than enhanced proliferation; may act as an EMT activator that decreases SOCS1 level and promotes STAT3 signaling.	High levels of miR-155-5p were associated with a poor prognosis.	[33]
Zahran et al., 2015	miR21, miR-184	OSCC	Salivary	80 subjects with HNSCC and 20 healthy controls	-	-	-	Diagnostic biomarkers for oral malignant transformation, There was a remarkable increase in salivary miRNA-21 and miRNA-184 in OSCC and potentially malignant disorders.	-	[34]
Ren et al., 2014	miR-21	tongue SCC	tumor and normal adjacent tissue	24 patients	Tca8113 and CAL-27	PDCD4	mitochondrial apoptosis pathway	Regulates chemo-sensitivity to cisplatin by targeting PDCD4. Inhibition of miR-21or PDCD4 can enhance or decrease cisplatin induced apoptosis, respectively, through modulation of mitochondrial apoptosis pathway.	-	[35]
Supic et al., 2018	miR-183 and miR-21	tongue SCC	fresh-frozen tissue of tongue carcinomas	60 patients	-	-	-	miR-183 and miR-21 in tumor tissue are markers of clinical stage and poor survival of patients and may be associated with high alcohol use.	-	[36]
Weng et al., 2017	miR-373-3p	tongue SCC	tumor and adjacent normal tissues	63 patients	SCC-9, SCC-15, SCC-25, UM1, UM2	DKK1	Wnt/β-Catenin Pathway	miR-373-3p targets DKK1 to increase EMT-associated metastasis.	-	[37]
Fu et al., 2017	MiR-155	OSCC	oral mucosa	46 cases of OSCC and 25 control	Tca8113 cells	p27Kip1	-	miR-155 induced cell cycle in G1 phase, weakened cell proliferation and blocked cell apoptosis.	-	[38]
Wang et al., 2017a	miR182	OSCC	tissues and adjacent noncancerous tissues	10 patients	Tca8113 cells	RASA1 and SPRED1	Ras-MEK-ERK pathway	miR-182 enhances cell proliferation and cell-cycle progression, inhibits OSCC cell apoptosis and enhances invasive capacity of OSCC cells.	-	[39]
Zhao et al., 2016	miR-24	tongue SCC	paired tumor and corresponding non-tumor control tissues	84 patients	UM1, UM2, Cal27, SCC1, SCC15 and SCC25	FBXW7	FBXW7 pathway	miR-24 enhances proliferation, migration and invasion.	-	[40]
Liu et al., 2018	MiR-1275	HNSCC	paired HNSCC and corresponding non-tumor control tissues	15 patients	PCI-4A/B and PCI-37A/B	IGF-1R and CCR7	PI3K/AKT pathway	miR-1275 promotes cell migration, invasion and proliferation.	-	[41]
Yeh et al., 2015	miR-372	HNSCC	paired HNSCC and corresponding non-tumor control tissues	66 patients	FaDu, OC3, OECM1, SAS and SCC25	p62	mTOR pathway	miR-372 promotes the migration of HNSCC cells by targeting p62.	-	[42]
Wong et al., 2008	miR-184	tongue SCC	paired tongue SCC and the normal tissues, and plasma	20 paired tongue SCC and the normal tissues, and plasma from 38 normal individuals and 30 tongue SCC patients	Cal27, HN21B, and HN96	-	-	miR-184 has antiapoptotic and proliferative effects.	-	[43]
Lu et al., 2012	miR-10b	OSCC	plasma samples	54 patients with oral cancer	SCC25, SAS, OECM1, OC3, CGHNC8, CGHNC9, CGHNK2, CGHNK4, CGK1, CGK5, CGK6	-	-	miR-10b increases cell migration and invasion. Plasma miR-10b may be a novel less-invasive biomarker for the early detection of oral cancer.	-	[44]
Tu et al., 2015	miR-372 and -373	OSCC	paired oral SCC and the normal tissues	50 patients	OECM-1, SAS and normal oral keratinocyte	LATS2	-	miR-372 and miR-373 enhance cancer cell migration and invasion in vitro and in vivo.	High miR372 and miR-373 expression associated with worse prognosis. tumor size, nodal status.	[45]
Sakha et al., 2016	miR-342–3p and miR-1246	OSCC	-	-	HOC313	DENND2D	-	miR-1246 increase cell motility but not cell growth	-	[46]
Zhuang et al., 2017	miR-218	OSCC	paired oral SCC and the normal tissues	61 patients	UM1, UM2, Cal27, MD1386Ln, SCC9, SCC15, SCC25, Tca8113	PPP2R5A	PPP2R5A/Wnt signaling pathway	miR-218 induced cell survival and resistance to cisplatin, and inhibits apoptosis by targeting PPP2R5A.	-	[47]
Du et al., 2017	miR-221	OSCC	-	-	SCC4 and SCC9	TIMP3	-	miR-221 protects cancer cells from apoptosis.	-	[48]
Zhou et al., 2016	miR-221/222	OSCC	-	-	293T, CAL27 and HSC6	PTEN	PI3K/Akt signaling pathway	miR-221/222 promotes cell proliferation, induces invasive ability of cells and inhibits cell apoptosis.	-	[49]
Zheng et al., 2015	miR-24	tongue SCC	paired tumor and normal tissues	79 patients	8 TSCC cell lines	PTEN	PTEN/Akt pathway	miR-24 induces cell survival, cell invasion and migration and cisplatin resistance through targeting PTEN.	-	[50]
Cheng et al., 2016	miR-455-5p	OSCC	paired tumor and normal tissues	40 patients	CGHNK2, OEC-M1, SCC15, TW2.6	UBE2B/TGF-β	TGF-β/SMAD pathway	miR-455-5p enhances the proliferation and growth of cells.		[51]
Guo et al., 2015	miR-96	tongue SCC	paired TSCC tissues and adjacent normal tissues	50 patients	Tca8113 and hNOK	MTSS1	-	miR-96 mediates cell proliferation and metastasis	-	[52]
Hu et al., 2016	miR-497	OSCC	resected specimens from OSCC patients	30 patients	SCC-15	SMAD7	-	miR-497 increases metastasis potential through SMAD7 suppression.	High level of miR-497 contributes to the distal metastases of primary OSCC and poor prognosis.	[53]
Kawakubo-Yasukochi et al., 2018	miR200c-3p	OSCC	-	-	SQUU-A and SQUU-B	CHD9 and WRN	-	miR200c-3p induces invasive potential in noninvasive cells.	-	[54]
Lu et al., 2019	miR-31-5p	OSCC	paired match tumor tissues and adjacent tissues, and sera	11 patients, sera from 82 oral cancer patients and 53 normal subjects	HaCaT, NOK-16B, SCC4, SCC9, SCC15, SCC25, CAL27, UM1/UM2, 1386Tu/1386Ln, 686Tu/686Ln	AKT and PTEN	PI3K/AKT pathway	miR-31-5p enhances the tumor growth and proliferation of oral cancer cells. miR-31-5p can be a diagnostic biomarker.	-	[55]
Jakob et al., 2019	miR-99b-3p	OSCC	paired match tumor tissues and adjacent tissues,	36 tumor tissue and 17 healthy oral mucosal tissue	-	-	-	hsa-mir-99b-3p plays a prognostic role in OSCC.	-	[56]
UMA MAHESWARI et al., 2020	miR-21 and miR-31	OPMD	salivary	36 healthy participants and 36 patients	-	-	-	miR-21 can be used as a potential diagnostic marker to evaluate very early malignant changes.	-	[57]
Shi et al., 2019	miR-626 and miR-5100	OSCC	Serum samples and tissue specimens	218 patients and 90 healthy controls	-	-	-	These miRNAs strongly relate to the prognosis for OSCC.	Higher expression of miR-626 and miR-5100 was correlated with poor outcome.	[58]
Hsing et al., 2019	miR-450a	OSCC	paired tumor and normal tissues	35 patients	DOK and SAS cells	TMEM182/TNFα	ERK and NFκB pathways	miR-450a mediates cellular adhesion and invasion in OSCC.	High level of miR-450a increased OSCC cells invasion ability.	[59]
Li et al., 2018a	miR-182-5p	OSCC	paired tumor and normal tissues	20 patients	hNOK, Tca8113, CAL-27, SCC-4, UM-1, and OSC-4	CAMK2N1	AKT, ERK1/2, and NF-κB pathways	miR-182-5p increases cell viability and promotes colony formation.	-	[60]
Lin et al., 2016b	miR-187	OSCC	paired tumor and normal tissues	56 patients and 19 control subjects	HSC3, OECM1, SAS, 293FT	BARX2	-	miR-187 increases oncogenicity and metastasis.	-	[61]
Liu et al., 2015	miR-92b	OSCC	paired tumor and normal tissues	85 patients	CAL-27, FaDu, SCC9, SCC25	NLK	NF-κB signaling pathway	miR-92b enhances cell proliferation and suppress the apoptosis.	-	[62]
Lu et al., 2018	miR-654-5p	OSCC	paired tumor and normal tissues	157 patients	Tca-8113, CAL-27	GRAP	Ras/MAPK Signaling pathway	miRNA-654-5p promotes proliferation, migration, invasion and chemoresistance by regulating EMT.	miR-654 expression was correlated with poor prognosis and lymph node metatstasis.	[63]
Peng et al., 2018	miR-134	OSCC	paired tumor and normal tissues	42 patients	OC3, FaDu, SCC25, HSC3, OECM1, SAS, 293T cells	PDCD7	-	miR-134 enhances OSCC progression by decreasing PDCD7 and E-cad expression.		[64]
Qiao et al., 2017b	miR-27a-3p	OSCC	-	-	SCC-9 and Tca8113	SFRP1	Wnt/β-catenin signaling pathway	miR-27a-3p stimulates EMT via the Wnt/β-catenin signaling pathway by targeting SFRP1.	-	[65]
Zheng et al., 2016	miR-21	Tongue SCC	paired tumor and normal tissues	44 patients	Tca8113, SCC-25 and CAL-27	CADM1/MYCN	-	miR-21 increases chemo-resistance via targeting CADM1.	Patients with high miR-21 and MYCN expression have a poorer overall survival.	[10]
Zhao et al., 2017	miR-24	Tongue	paired tumor and normal tissues	90 patients	-	PTEN	-	miR-24 was associated with tumor progression.	Clinical stage, differentiation, miR-24 level, and PTEN expression level were correlated with prognosis.	[66]
Chen et al., 2016	miR-211	OSCC	paired tumor and normal tissues	50 patients	SAS, OECM1, HSC3, FaDu, OC4, and SCC25; 293T	TCF12	4NQO-miR-211-TCF12-FAM213A cascade	miR-211 is a regulator of OSCC by targeting the TCF12 tumor suppressor.		[67]
AHMAD et al., 2019	miR-15b-5p	HNSCC (oropharyngeal, laryngeal and hypopharyngeal carcinomas)	paired tumor and normal tissues	51 patients	-	-	-	miR-15b-5p is a biomarker for radiation response.	-	[68]
Hu et al., 2019	miR-196a	Esophageal SCC	tumor tissues and adjacent non-tumor tissues	25 patients with ESCC	Het-1A and EC109	ANXA1	-	miR-196a promotes the proliferation, invasion and migration by targeting ANXA1.	-	[69]
González-Arriagada et al., 2018	miR-26 and miR-125b	HNSCC (oral, oropharyngeal, laryngeal)	tumor tissues	16 primary HNSCC with lymph node metastasis 16 their matched lymph node, without metastasis	-	-	-	miR-26 and miR-125b may be related to the progression and metastasis.	-	[70]
Zhao et al., 2018	miR-196b	Laryngeal SCC	tumor tissues and normal laryngeal mucosa samples	113 tumor tissues from patients and 34 normal laryngeal mucosa samples	TU212 and TU177	SOCS2	-	miR-196b promotes cells proliferation and invasion, and precludes apoptosis.	miR-196b expression was an independent prognostic factors of overall survival.	[71]

HNSCC: head and neck squamous cell carcinoma, OSCC: oral squamous cell carcinoma, OPMD: oral potentially malignant disorder.

Table 1 displays the function and prognostic implication of onco-miRs in HNSCC.

## 3 Tumor suppressor miRNAs in HNSCC

Subsequently, we extracted data of 88 articles which demonstrated down-regulation of miRNAs in tissue/plasma samples of patients with HNSCC compared with controls. Potential targets of these miRNAs have been identified through in silico analyses or functional studies in the original articles. These miRNAs mostly regulate expression of pro-proliferative or anti-apoptotic genes. Lo et al. have shown down-regulation of miR-200c in the regional metastatic lymph node of HNSCC tissues, while *BMI1* expression was up-regulated as compared to parental tumors. Their functional investigations demonstrated direct interaction between miR-200c and the 3′ UTR of *BMI1* in HNSCC cells. They also reported down-regulation of this miRNA in isolated HNSCC-derived ALDH1^+^/CD44^+^ cells which had cancer stem cell (CSC) features. Forced over-expression of miR-200c could suppress the malignant CSC-like features of these cells. Notably, miR-200c over-expression decreased expressions of *ZEB1*, Snail and N-cadherin, while increased E-cadherin expression in ALDH1^+^/CD44^+^ cells. The role of miR-200c up-regulation in decreasing malignant phenotype was verified in a xenograft model as well [72]. Using next generation sequencing (NGS) technique, Allen et al. have studied the effect of serum from HNSCC patients on expression of miRNA in exposed cells in vitro. Their results showed induction of a specific miRNA expression profile in the exposed cells following treatment with patients' serum samples. The analysis of gene ontologies and pathway analysis showed involvement of these miRNAs in cancer-related pathways such as cell cycle and apoptosis. Most importantly, P53 and SLC2A1 were direct targets of these miRNAs [73]. Table 2 displays the list of down-regulated miRNAs in HNSCC samples and their functions.

**Table 2:** List of down-regulated miRNAs in HNSCC.

Author/year	microRNA	Cancer subtype	Tissues	Numbers of clinical samples	Assessed cell line	Targets/Regulators	Signaling Pathways	Function	Patient's prognosis	Ref
Kalfert et al., 2015	miR-375	HNSCC (oropharyngeal, laryngeal and hypopharyngeal carcinomas)	paired tumor and control tissue	51 patients	-	-	-	Down-regulated in oropharyngeal, laryngeal and hypopharyngeal carcinomas, potential prognostic significance	-	[9]
Childs et al., 2009	miR-205, let-7d	HNSCC	paired tumor and control tissues	104 patients	-	DHFR, KRAS	P53	miR-205 and let-7 could prevent tumor growth by negatively regulating DHFR and p53 pathway as well as KRAS.	Shorter time to death and loco-regional recurrence in patients who have combined lower absolute levels for miR-205 and let-7d.	[12]
Lo et al., 2011	miR-200c	HNSCC	paired tumor and control tissue	five patients	Isolated ALDH1+CD44 + cell subsets from HNSCC tissue from five patients	BMI1	ZEB1 and ZEB2 pathways in EMT signaling	miR200c inhibits self-renewal, radioresistance, high *in vivo* tumorigenicity, invasion, and distant metastasis in ALDH1+/CD44 + HNSCCs by negatively modulating *BMI1*.	-	[72]
Lubov et al., 2017	miR-17, miR-26, miR-29, miR-31, miR-34, miR-125, miR-126, miR-137, miR-138, miR-143, miR-152, miR-200, miR-203, miR-205, miR-206, miR-218, miR-324, miR-363, miR-375, miR-451, miR-489, miR-491, miR-506, miR-519, miR-639, and let-7d	HNSCC	paired tumor and control tissue	A meta-analysis study includes 8,194 subjects with HNSCC	-	-	apoptotic and cell death signaling pathways	Decreased expressions of these miRNAs were correlated with lower survival and metastasis in HNSCC.	Decreased expressions of these miRNAs were correlated with lower survival and metastasis.	[14]
Hou et al., 2015	miR-99a	HNSCC	paired tumor and control tissue and plasma	16 paired tissue samples from patients with HNSCC and 9 paired plasma samples prior and 6 months after surgical removal of tumor	-	MTMR3, IGF1R, mTOR, SMARCA5	-	Dysregulation of circulating miR-99a is involved in the therapeutic response.	-	[15]
Kuo et al., 2014	miR-99a	oral cancer	paired tumor and normal tissues	26 patients	NOKs, DOK, CAL-27, SCC-9, SCC-15, SCC-25, OC-2, OC-3, OEC-M1, HSC-3, HMEC-1	MTMR3	-	miR-99a has anti-metastatic effect.	-	[74]
Greither et al., 2017	miR-93 and miR-200a	HNSCC (oropharyngeal, oral, laryngeal squamous cell carcinoma)	saliva samples	83 saliva samples from 33 patients collected at numerous times pre-, during and post-radiotherapy treatment.	-	ZEB2 and CTNNB1	-	Biomarkers for the treatment monitoring post-radiation of HNSCC	-	[75]
Yen et al., 2014	miR-99a	OSCC	paired tumor and normal tissues	40 patients	CGHNC9, OC3, OEC-M1, TW2.6, FaDu, KB, SCC-4, SCC15, SCC9, SCC25, UT-MUC-1, YD-15, DOK, Tu183, UMSCC1, HSC3	IGF1R	PI3K/IGF1R signaling	miR-99a acts as a metastasis suppressor and regulates IGF1R expression.	-	[76]
Yuan et al., 2019	microRNA-545	OSCC	paired tumor and normal tissues	20 patients	HSC2, HSC4, SAS, KON	RIG-I	human papilloma virus (HPV) infection pathway	tumor suppressive role of miR-545 in OSCC	-	[77]
Hudcova et al., 2016	hsa-miR-375-3p	HNSCC	biopsy samples of tumors from male patients	42 patients	-	-	-	Diagnostic marker in HNSCC	-	[78]
Hudcova et al., 2016	hsa-miR-29c-3p	HNSCC		42 patients	-	-	-	Down-regulation of hsa-miR-29c-3p in tumor tissue was associated with higher tumor grade. Down-regulation of hsa-miR-29c-3p in tumor-adjacent tissue was associated with worse overall and disease-specific survivals.	-	[78]
Hudcova et al., 2016	hsa-miR-200b-5p	HNSCC		42 patients	-	-	-	Down-regulation of hsa-miR-200b-5p in tumor tissue was significantly associated with higher tumor grade.	-	[78]
Manikandan et al., 2016	let-7a	OSCC	tumor and normal tissues	discovery cohort (n = 29), validation cohort (n = 61)	-	BCL2, HMGA2, MYC, HRAS, and KRAS	PI3K/Akt signaling pathway	Inhibits cell proliferation, promoting apoptosis	-	[20]
Ren et al., 2020	miR-7109-5p	OSCC	tumor and normal tissues	six metastatic tumor samples, six nonmetastatic tumor samples, and six normal tissues	-	MMP7	TGF-beta signalling pathway	promising prognostic and diagnostic indicator or potential cancer therapeutic target	-	[79]
Ren et al., 2020	miR-34b	OSCC	tumor and normal tissues	six metastatic tumor samples, six nonmetastatic tumor samples, and six normal tissues	-	MMP13	TGF-beta signaling pathway	Prognostic and diagnostic indicator or potential cancer therapeutic target	-	[79]
Allen et al., 2018	miR-32-5p^&^	HNSCC (oropharyngeal, oral, laryngeal squamous cell carcinoma)	serum samples	7 HNSCC patients and 4 healthy individuals	HeLa	MDM2, Sirt1	P53 pathway	Down-regulation of this miRNA could enhance p53 inhibition in the treated cells.	-	[73]
Allen et al., 2018	miR-128-3p^&^	HNSCC (oropharyngeal, oral, laryngeal squamous cell carcinoma)	serum samples		HeLa	Sirt1	P53 pathway	Reduced expression of this miRNA could facilitate p53 inhibition in the treated cells.	-	[73]
Allen et al., 2018	miR-212-5p^&^	HNSCC (oropharyngeal, oral, laryngeal squamous cell carcinoma)	serum samples		HeLa	CCND1	Cell cycle	miR-212-5p targets CCND1.	-	[73]
Allen et al., 2018	miR-132-5p^&^	HNSCC(oropharyngeal, oral, laryngeal squamous cell carcinoma)	serum samples	7 HNSCC patients and 4 healthy individuals	HeLa	Bcl2	apoptosis	miR-132-5p targets Bcl2. Bcl2 suppresses apoptosis.	-	[73]
Salazar-Ruales et al., 2018	miR-92a-3p, miR-124-3p, and miR-205-5p	HNSCC	saliva samples	108 cases and 108 controls	-	-	-	Biomarkers in HNSCC, with high sensitivity and specificity	-	[24]
Hauser et al., 2015	miR-128	HNSCC	-	-	JHU-13, JHU-22	BMI-1, BAG-2, BAX, H3f3b, Paip2	proliferation and apoptotic pathways	miR-128 has a tumor suppressor function.	-	[80]
Hu et al., 2015	mir-375	laryngeal SCC	primary tumors and non- cancerous tissues	46 patients	-	phosphoinositide-dependent protein kinase-1	AKT pathway	Activates apoptosis by inhibiting anti-apoptotic AKT protein	-	[25]
Yan et al., 2017	miR-375, miR-92b-3p	OSCC	plasma samples	20 plasma samples obtained before, and 9–12 months after surgical removal of the tumor, and 18 healthy controls	-	-	-	help monitoring OSCC recurrence following surgery	-	[81]
Yan et al., 2017	miR-486-5p	OSCC	plasma samples	20 plasma samples obtained before, and 9–12 months after surgical removal of the tumor, and 18 healthy controls	-	ARHGAP5	insulin growth factor signaling	Circulating miR-486-5p are biomarker for OSCC diagnosis and recurrence after surgery.	-	[81]
Victoria Martinez et al., 2015	miR-26a/b	HNSCC(oral, oropharyngeal, laryngeal)	Serum	7 males with HNSCC and 7 healthy control males	-	cyclin D2	-	Suppression of cell proliferation, induction of tumor-specific apoptosis, and protection from disease progression	-	[26]
Victoria Martinez et al., 2015	miR-150	HNSCC(oral, oropharyngeal, laryngeal)	Serum	7 males with HNSCC and 7 healthy control males	-	PIM1 and EP300	-	Regulation of cell growth and division	-	[26]
Victoria Martinez et al., 2015	miR-98	HNSCC(oral, oropharyngeal, laryngeal)	Serum	7 males with HNSCC and 7 healthy control males	-	-	-	Regulation of tumor metastasis	-	[26]
Ries et al., 2017	miR-186	OSCC	Whole blood	21 patients with recurrence of OSCC and 21 patients without recurrence	-	-	-	Useful in prognostic applications	-	[27]
Wang et al., 2017d	miR-195-5p	OSCC	paired tumor and normal oral epithelial tissues	40 patients	Tca83 and Cal27	TRIM14	NF-κB signaling pathway	Its overexpression promotes apoptosis and reduces cell growth, migration, and invasion.	-	[82]
Zhang et al., 2017	miR-375	OSCC	paired tumor and normal oral epithelial tissues	44 patients	SCC-4	IGF-1R	IGF-1R, signaling pathway, MAPK and pathways	Overexpression of miR-375 suppresses growth, induces cell cycle arrest in G0/G1 phase, induces apoptosis and increases radiosensitivity in OSCC cells, and it is potential therapeutic target.	-	[83]
Feng et al., 2019	miR-532-3p	tongue squamous cell carcinoma (TSCC)	paired tumor and paratumor tissues	23 patients	TSCCA, TCA8113, CAL-27, and SCC-25	CCR7	-	Up-regulation of miR-532-3p inhibits cell proliferation, migration, invasion, and induces apoptosis.	-	[84]
Harrandah et al., 2016	miR-375 and miR-494	OSCC	paired tumor and non-tumor tissues	31 samples from progressive premalignant lesions and paired sequential OSCC tumors	-	-	-	is associated with a higher risk of malignant transformation	-	[85]
Shi et al., 2018	miR-488	TSCC	paired tumor and non-tumor tissues	20 TSCC tissues and 10 their adjacent non-cancer tissues	UM1, TCA8113, Cal27, SCC1 and SCC25	ATF3	SAPK/JNK stress pathway	miR-488 suppresses cell invasion and EMT by direct downregulation of ATF3.	-	[86]
Shang et al., 2018	miR-9	OSCC	tumor tissues and adjacent normal tissues	21 patients	Tca8113	CDK4/6	G1/S transition pathway	miR-9 decreases cell proliferation and migration.	-	[87]
Lin et al., 2017	miR-485-5p	OSCC	-	-	SCC25 and SCC25-res	PAK1		miR-485-5p reverses EMT and enhances cisplatin-induced cell death by targeting PAK1, and significantly inhibited invasion and migration in oral tongue squamous cell carcinoma.	-	[88]
Yu et al., 2011	let-7a	HNCC	-	-	HNC-ALDH1 (+) cells relative to HNC-ALDH1(-) cells	Nanog/Oct4	-	let-7 suppresses tumor metastasis and improves survival time.	-	[89]
Alajez et al., 2012	let-7a	HNSCC (laryngeal and hypopharyngeal carcinomas)	tumor tissues and adjacent normal tissues	Paired tissues from 20 patients with recurrence and 19 patients without recurrence	NOE and HNSCC FaDu	HMGA2, CCND2, IGF1R, and IGF2BP2/Lin28b as regulator of let-7	IGF pathway	Regulation of the IGF pro-survival pathway	-	[90]
RIES et al., 2014	miR-186	OSCC	Whole blood	50 patients and 33 controls	-	-	-	Induces cellular senescence and regulate apoptotic response	-	[29]
Lu et al., 2011	miR-26a	nasopharyngeal carcinoma	tumor tissues and normal tissues	18 tumor samples and 16 normal nasopharyngeal epithelial tissues	NP69, 5–8F, 6–10B, CNE1, CNE2, C666-1, HONE1, and HNE-1, HEK 293T	EZH2, CCND2	-	miR-26a inhibited cell growth partly due to a G1-phase arrest	-	[91]
Tang et al., 2014	miR-205-5p, miR-135b-5p	Nasopharyngeal Carcinoma	tumor tissues and normal tissues	67 fresh NPC and 25 normal control tissues	-	-	-	Diagnostic value	-	[92]
Koshizuka et al., 2017	miR-199a-5p, miR-199a-3p, miR-199b-5p, miR-199b-3p	HNSCC (floor of the mouth and tongue)	tumor tissues and normal tissues	22 tissue specimens from patients with HNSCC and 22 normal epithelial tissues	SAS and HSC3	ITGA3, PXN	focal adhesion pathway	miR-199 family suppresses cell migration and invasion.	-	[93]
Islam et al., 2014	miR-138	HNSCC(floor of the mouth and tongue)	primary tumors	18 patients	UM-SCC-1 and -47	RhoC, FAK, Src and Erk(1/2)	Erk1/2 signaling pathway	miR-138 is a tumor suppressor miRNA that reduces cell motility, colony and stress fiber formation.	-	[94]
Kinoshita et al., 2013	miR-29s	HNSCC (Tongue, Oral floor, Oropharynx, Larynx and Hypopharynx)	paired tumor and normal tissues	23 patients	SAS and FaDu	LAMC2 and ITGA6	focal adhesion pathway	miR-29s suppresses cancer cell migration and invasion by targeting laminin–integrin signaling.	-	[95]
Shiah et al., 2014	miR-329 and miR-410	OSCC	Paired tumor specimens and their adjacent nontumorous epithelia	40 patients	DOK, FaDu, OC-3, OEC-M1, SCC-4, SCC-9, SCC-15, SCC-25, Tw2.6, and YD-15	Wnt-7b	Wnt signaling pathway	miR329 and miR410 inhibit the proliferation and invasion by targeting Wnt-7b.	-	[96]
Chang et al., 2016	miR-376c	HNSCC	paired tumor and normal samples	40 patients	HOKs, 293T, Cal-27, Ca9-22 and SAS	RUNX2	RUNX2/INHBA axis	miR-376c suppresses lymph node metastasis by RUNX2/Activin-A axis.	Low miR-376c-3p levels predict poor prognosis in HNSCC.	[97]
Xu et al., 2015a	miR-143	OSCC	paired tumor and normal samples	49 patients	SCC-4, Tca-8113, CAL-27	CD44 v3	phospho-c-Met signal pathway	miR-143 suppresses migration and invasion.	-	[98]
Zahran et al., 2015	miR-145	OSCC	Saliva	80 subjects with HNSCC and 20 healthy controls	-	-	-	noninvasive, rapid diagnostic biomarkers for oral malignant transformation	-	[34]
Cao et al., 2015	miR-26b	Tongue SCC	tissues of tongue SCC and the matched normal counterparts	30 patients	HSC-3, SCC-4, Cal27, hNOKs	PTGS2 (COX2)	VEGF-C pathway	miR-26b serves as a tumor suppressor by targeting COX-2.	Low miR-26b expression is correlated with advanced clinical stage, lymph node metastasis, and poor prognosis.	[99]
Wu et al., 2017a	miR-802	Tongue SCC	paired tumor and normal samples	20 patients	SCC1, SCC4, Cal27 and UM1	MAP2K4	MAPK signaling pathway	miR-802 suppresses cell viability and invasion through targeting MAP2K4.	-	[100]
Sun et al., 2016a	miR-137	Tongue SCC	paired tumor and normal samples	25 patients	SCC4, SCC1, UM1 and Cal27	SP1	-	miR-137 suppressed TSCC cell proliferation, colony formation, EMT cell invasion and migration	-	[101]
Wang et al., 2016	miR-204-5p	OSCC	frozen OSCC patient specimens	52 patients	Human OSCC cell lines	CXCR4	Wnt/b-catenin and NF-kappaB signaling pathways	miR-204-5p suppressed OSCC cellular growth and metastasis.	-	[102]
Sun et al., 2016b	miR-9	OSCC	Serum	104 OSCC patients, 30 OLK patients, and 40 healthy volunteers	-	-	-	Serum miR-9 is an independent risk factor for OSCC.	Low miR-9 expression level predicts poor prognosis.	[103]
Yang et al., 2017	miR-381-3p	OSCC	tumor specimens and adjacent tissue	18 patients	SCC-9, Tca-8113	FGFR2	-	miR-381-3p suppresses cell proliferation and enhances apoptosis by directly targeting FGFR2.	-	[104]
Hashiguchi et al., 2018	miR-205	OSCC	-	-	HSC-2, HSC-3, SQUU-A, SQUU-B, SQUU-BO, SQUU-BC, SAS	ZEB1 or ZEB2	EMT signaling pathway	miR-205 contribute to EMT suppression.	-	[105]
Shi et al., 2015a	miR-375	OSCC	paired tumor and adjacent non-tumorous mucosa specimens	17 patients	CAL27, WSU-HN6, HEK-293T	KLF5	-	miR-375 can suppress cellular proliferation and induce cell apoptosis.	-	[106]
Wu et al., 2017b	miR-375	OSCC	paired tumor and adjacent non-tumorous mucosa specimens	40 patients	Hs 680.Tg, Fadu, SCC-25, CAL-27 and Tca8113	SLC7A11	-	miR-375 suppresses proliferation and invasion through suppressing the expression of SLC7A11.	-	[107]
Ji et al., 2017	miR-138	Tongue SCC	tumor samples and normal tissues	137 tumor samples and 20 normal tongue tissues	UM1 and UM2	AKT1	AKT/ERK1/2 pathway	miR-138 directly targets AKT1 and decreases the invasion and metastatic potential of TSCC cells	Low miR-138 levels predict poor prognosis.	[108]
Xu et al., 2015b	miR-138	OSCC	paired tumor and normal tissues	20 patients	OC3, KB, OEC-M1, HSC3 and SCC-4	YAP1	Hippo pathway	miR-138 suppresses the proliferation and growth of OSCC by targeting YAP1.	-	[109]
Kim et al., 2018	MiR-203	OSCC	-	-	YD-38 cells and normal human oral keratinocytes	Bmi-1	-	miR-203 decreases the viability of YD-38 cells and significantly induces apoptosis by directly targeting Bmi-1.	-	[110]
Lim et al., 2017	miR-203	OSCC	-	-	YD-38 cells and normal human oral keratinocytes	SEMA6A	-	miR-203 reduces the viability of YD-38 cells and activated the apoptotic signaling pathway	-	[111]
Lin et al., 2016a	miR-203	Tongue SCC	paired tumor and adjacent non-tumorous specimens	10 patients	Tca8113	PIK3CA	-	miR-203 induces a cell cycle arrest and increases the apoptotic	-	[112]
Xie et al., 2018	miR-200c	OSCC	paired tumor and normal tissues	32 patients	HOC313	ZEB1	EMT pathway	miR-200c significantly suppressed cell invasion and migration, and suppressed EMT via negatively regulating ZEB1 expression.	-	[113]
Zhao et al., 2015	miR-222	Tongue SCC	tissue samples for primary cultural cells	6 patients	UM1 and UM2	ABCG2/ERCC1	-	miR-222 inhibits migratory/invasive potential.	-	[114]
Wang et al., 2017e	miR-15b	tongue SCC	-	-	SCC25 and SCC25-res cells	TRIM14	-	miR-15b inhibits TRIM14 and suppresses cancer-initiating cell phenotypes, and enhances MET thus sensitizing cisplatin-resistant SCC25 cells to cisplatin.	-	[115]
Li et al., 2017	MiR-124	OSCC	paired tumor and normal tissues	6 patients	SCC-9 and CAL-27	CCL2 and IL-8	-	miR-124 suppresses tumor growth.	-	[116]
Lin et al., 2014	miR-639	tongue SCC	paired tumor and adjacent non-tumorous specimens	92 patients	SCC9 and CAL27	FOXC1	TGFβ-induced EMT pathway	Inhibits TGFβ-induced EMT	Low levels of miR-639 correlate with lymph node metastasis and poor prognosis.	[117]
Liu et al., 2017	miR-27b	OSCC	-	-	Tca8113 and SCC-4	FZD7	Wnt signaling pathway	mR-27b suppresses cell proliferation by targeting FZD7 and Wnt signaling pathway.	-	[118]
Min et al., 2016	miR-148a	OSCC	-	-	NFs and CAFs were isolated from OSCC tumor tissues and SCC-25 cells	Wnt10b	-	miR-148a decreased the migration and invasion through targeting WNT10B mediated signal pathway.	-	[119]
Qiao et al., 2017a	MicroRNA-542-3p	OSCC	paired tumor and adjacent non-tumorous specimens	108 patients	CRL-1629	ILK, TGF-β1 and Smad2/3	ILK/TGF-β1/Smad2/3 signaling	miR-542-3p inhibits self-renewal, invasiveness, migration, proliferation and survival.	Low level of miR-542-3p indicated poor prognosis.	[120]
Qiu et al., 2016	miR-22	tongue SCC	-	-	TCA8113 cells	CD147	-	miR-22 inhibited cell proliferation and motility and down-regulated CD147.	-	[121]
Rastogi et al., 2017	miR-377	OSCC	tissues	20 patients	UPCI-SCC-116	HDAC9	HDAC9/NR4A1/Nur77 pathway	miR-377 inhibits cell growth, induces apoptosis, and decreases cell migration.	-	[122]
Ruan et al., 2018	miR-30a-5p	OSCC	oral cancer tissues and adjacent normal tissues	66 oral cancer tissues and 25 adjacent normal tissues	NHOECs, SCC-15, SCC-25, SCC-4, Tca-8113 and HEK-293T	FAP	Ras-ERK	miR-30a-5p suppresses the cell proliferation, the migration and invasion of oral cancer cells via down-regulating FAP.	-	[123]
Jia et al., 2020	MiR-148a	OSCC	paired tumor and normal tissues	110 patients	SCC-15 and HOK	IGF-IR	ERK/MAPK pathway	miR-148a suppresses OSCC cell proliferation, migration and invasion by targeting IGF-IR and suppressing ERK/MAPK signaling pathway.	-	[124]
Shi et al., 2015b	miR-146a	OSCC	oral carcinoma tissues and adjacent normal tissues	40 oral squamous cell carcinoma tissues and 10 adjacent normal oral mucosa tissues	SCC25 and UMSCC1	Sox2	-	Inhibits tumor aggressiveness	-	[125]
Wang and Liu, 2016	miR-188	OSCC	paired tumor and normal tissues	22 patients	KB, FaDu, and Detroit 562	SIX1	cyclin D1/MMP9/p-ERK pathway	miR-188 is a tumor suppressor and suppresses proliferation and invasion by targeting SIX1.	-	[126]
Wang et al., 2017b	miR-139-5p	OSCC	paired tumor and normal tissues	40 patients	NOK, SAS, TCA8113, KON	HOXA9	-	miR-139-5p suppresses the viability, proliferation, invasion and migration.	-	[127]
Wang et al., 2017c	miR-376c-3p regulates	OSCC	paired tumor and normal tissues	49 patients	SCC-4, SCC-9, SCC-15, SCC-25 OSCC	HOXB7	-	miR-376c-3p inhibits proliferation, viability, migration and invasion and induces G1/G0 arrest and cell apoptosis.	-	[128]
Wang et al., 2018a	miR-655	OSCC	paired tumor and normal tissues	26 patients	Tca8113, CAL-27 and SCC-9	MTDH	PTEN/AKT pathway	miR-655 suppresses cell proliferation and invasion by targeting MTDH.	-	[129]
Wang et al., 2018c	miR-1294	OSCC	paired tumor and normal tissues	24 patients	HSC2, HSC4, SAS and KON	c-Myc, TRL4, TLR6, TLR8 and TLR9	-	miR-1294 inhibited cell growth and cell migration.	-	[130]
Weng et al., 2016	miR-494-3p	OSCC	paired tumor and normal tissues	45 patients	SAS cells	Bmi1	-	miR-494-3p induces cellular senescence and enhance radiosensitivity.	-	[131]
Chang et al., 2015	miR-494	HNSCC	pairs of tumor and adjacent noncancerous matched tissues	45 patients	S-G human gingival epithelial cell line, SAS	Bmi1 and ADAM10	-	miR-494 inhibits tumor aggressiveness.	-	[132]
Xu et al., 2016b	miR-340	OSCC	paired tumor and normal tissues	3 patients	SAS and HEK293 T cells	Glut1		miR-340 inhibits growth, induces a metabolic shift		[133]
Zeng et al., 2016	miR-27a-3p	OSCC	paired tumor and normal tissues	50 patients	Tca8113, CAL-27, SCC-4, SCC-9, SCC-25, HN-6 and hNOK	YAP1	YAP1-OCT4-Sox2 signal axis	miR-27a-3p downregulates the EMT-related molecules effectively and suppress EMT process, invasion and metastasis.	-	[134]
Li et al., 2018b	miR-218-5p	OSCC	-	-	UM-SCC6	CD44	CD44-ROCK pathway	miR-218-5p suppresses cell invasion.	-	[135]
Gao et al., 2019	miR-145-5p	Laryngeal SCC	paired tumor and normal tissues	40 patients	human 293T and LSCC cell line Hep-2, TU177	FSCN1	EMT pathway	miR-145-5p inhibites migration, invasion, and growth by suppressing EMT.	Low miR-145-5p/high FSCN1 levels predict poor prognosis.	[136]
Chou et al., 2019	miR-486-3p	OSCC	paired tumor and normal tissues	46 patients	OKF4/hTERT, OEC-M1 and TW2.6 OSCC	DDR1/ANK1	-	miR-486-3p inhibits proliferation and activates apoptosis.	-	[137]
Jakob et al., 2019	mir-100-5p	OSCC	tumor tissue and healthy oral mucosal tissue	36 tumor tissue and 17 healthy oral mucosal tissue	-	-	-	prognostic impact	-	[56]
Ding et al., 2019	miR-145	OSCC	tumor tissues and adjacent normal tissues.	48 patients	SCC-9	HOXA1	ERK/MAPK signaling pathway	miR-145 inhibits cell viability, invasion, and migration.	-	[138]
Cao et al., 2017	MiR-375	OSCC	-	-	Tca8113, UM2, UM1 and CAL-27	PDGF-A	-	miR-375 suppresses the migration and invasion of OSCC.	-	[139]
Du et al., 2015	miR-98	OSCC	paired tumor and normal tissues	19 patients	SCC-25 and Tca-8113	IGF1R	-	miR-98 inhibits tumor cell growth and metastasis by targeting IGF1R.	-	[140]
Fadhil et al., 2020	miR-let-7a-5p and miR-3928	HNSCC (glottis, buccal sulcus, buccal mucosa, tongue, and floor of the mouth (FOM))	saliva	150 HNSCC patients and 80 healthy controls	-	-	-	Biomarkers for diagnosis and prognostic indicators	-	[141]
Hersi et al., 2018	miR-9	HNSCC	-	-	H357, HN5, HN30, HEK293T, HSC3 and HSC3M3	CXCR4	-	miR-9 expression has a significant tumor suppressor role in HNSCC cells, potentially through inhibition of cellular proliferation, cell cycle progression, migration and colony formation.	-	[142]
González-Arriagada et al., 2018	miR-203	HNSCC (oral, oropharyngeal, laryngeal)	primary HNSCC with lymph node metastasis and their matched lymph node, without metastasis	16 primary HNSCC with lymph node metastasis 16 their matched lymph node, without metastasis	-	-	-	-	miR-203 is associated with good prognosis.	[70]
Wang et al., 2018b	miR-200	OSCC	-	-	SCC25 and SCC15	EZH2	STAT3 signaling pathway	miR-200 mediates antitumor functions by targeting STAT3 signaling.	-	[143]
Kang et al., 2018	miR-300	OSCC	specimens of OSCC	120 patients	Tca8113, Cal-27 and HaCat	-	-	miR-300 could suppress metastasis by inhibiting EMT.	-	[144]
Dong et al., 2018	miR-876-5p	HNSCC (Buccal, Palate, Gingiva, Oropharynx, Tongue)	tumor tissues	40 patients	CAL27, HEK293T, WSU-HN4, WSU-HN6	vimentin	-	miR-876-5p inhibits cell migration and invasion.	-	[145]

HNSCC: head and neck squamous cell carcinoma, OSCC: oral squamous cell carcinoma, OPMD: oral potentially malignant disorder, ^&^: These miRNAs are down-regulated in cells treated with serum samples of patients with HNSCC.

## 4 Association with therapeutic response

A number of studies have assessed associations between expression amounts of miRNAs and patients' response to chemotherapeutic agents. For instance, Hebert et al. have shown that transfection of pre-miR-98™ into HNSCC cells during normoxia decreases expression of HMGA2. As HMGA2 expression promotes selective sensitivity to the topoisomerase II inhibitor, miR-98 confers resistance to doxorubicin and cisplatin [13]. Ren et al. have shown that transfection of miR-21 inhibitor into the tongue SCC cells enhances sensitivity to cisplatin. miR-21 inhibitor also enhances PDCD4 protein level as well. Besides, inhibition of miR-21 or PDCD4 could remarkably increase or decrease cisplatin-induced apoptosis, respectively. Thus, miR-21 has been suggested as a critical factor in modulation of chemosensitivity to cisplatin [35]. Table 3 shows the list of miRNAs that modulate response to doxorubicin or cisplatin.

**Table 3:** Role of miRNAs in chemoresistance in HNSCC based on up-/down-regulation of miRNAs.

Response to chemotherapeutic drug	miRNA	Reference
Doxorubicin resistance	miR-98 (up), miR-221 (up)	[13,48]
Cisplatin resistance	miR-21 (up), miR-203 (down), miR-222 (down), miR-15b (down), miR-218 (up), miR-98 (up), miR-24 (up), miRNA-654-5p (up)	[10,13,47,50,63,112,114,115]

Moreover, miRNA profiles can also predict response of patients to radiotherapy. Chen et al. have retrieved expression profile of 56 differentially expressed miRNAs between HNSCC tumors and adjacent normal specimens from the Cancer Genome Atlas (TCGA). Then, they compared expression of miRNAs in HNSCC patients getting adjuvant radiotherapy in relation with clinical outcomes. Based on the expression profile of five miRNAs namely miR-99a, miR-31, miR-410, miR-424, and miR-495, authors recognized that only low-risk group would profit from radiotherapy [146]. MiRNAs might also modulate response to targeted therapies such as monoclonal antibodies. Bozec et al. have shown that over-expression of miR-223 in SCC cells not only makes these cells more resistant to cisplatin, docetaxel, and 5-fluorouracil but also aggravates their response to the anti-EGFR monoclonal antibody cetuximab. This observation implies that expression of miR-223-3p enhances resistance to anticancer modalities [147].

## 5 Diagnostic/prognostic value of miRNAs in HNSCC

Recent studies have shown diagnostic value of miRNAs in HNSCC. They mostly designed receiver operating characteristic (ROC) curves and measured the area under curve (AUC) values to estimate diagnostic power of miRNAs. Such assessments have been accomplished in different biological sources such as saliva, whole blood, serum, and tumor tissue samples. Control samples were obtained from cancer-free individuals except for the latter in which paired non-tumoral samples from the same patients were used as controls. Moreover, a number of studies have assessed power of miRNAs in the differentiation between patients with recurrence and those without recurrence. Although all assays are practically useful, serum, blood and saliva provide non-invasive sources for diagnosis of cancer. Theoretically, miRNAs can be used for early/routine diagnosis of HNSCC as well as patients' follow-up for observation of relapses. Notably, miRNA signature can even discriminate different stages of HNSCC tumors [24]. Moreover, higher expression of several oncomiRs and lower expression of a number of tumor suppressor miRNAs were correlated with poor patients’ outcome as defined by disease free survival or overall survival. The predictive values of several miRNAs were also assessed in relation with clinicopathological factors such as grade, stage or the p16 status. Table 4 shows the results of studies that investigated this issue in HNSCC. Prognostic value of miRNAs was estimated through Kaplan-Meier or Cox regression evaluation.

**Table 4:** Summary of results of studies which investigated diagnostic/prognostic value of miRNAs in HNSCC.

Sample number	Area under curve	Sensitivity	Specificity	Kaplan-Meier analysis	Univariate cox regression	Multivariate cox regression	Reference
Forty samples of OSCC and 40 matched normal tissues	0.9	88%	99%	Higher levels of miR-191 suggesting a lower survival probability.	-	-	[17]
Salivary from 45 patients with OSCC and 24 controls	0.82 for miR-31	80%	68%	-	-	-	[21]
Saliva samples from 108 HNSCC cases and 108 controls	0.73 for miR-122-5p and 0.70 for miR-92a-3p	-	-	-	-	-	[24]
46 laryngeal SCC tumors and non- cancerous tissues	0.753 for miR-223/miR-375, 0.991 for miR-21/miR-375 and 0.856 for miR-142-3p/miR-375	-	-	High expression of miR-21/miR-375 in cancerous tissue associates with poor prognosis.	-	-	[25]
Whole blood of 21 patients with recurrence of OSCC and 21 patients without recurrence	0.80 for miR-3651 0.78 for miR-494 0.76 for miR-186	81% for miR-3651, 71.4% for miR-494, 71.4% for miR-186	71.4% for miR-3651, 76.2% for miR-494, 81% for miR-186	-	-	-	[27]
Whole blood of 50 OSCC patients and 33 controls	0.82 for miR-3651 0.72 for miR-494	-	-	-	-	-	[29]
20 saliva samples and 46 tissue samples from patients with OPMD	0.81	87.51%	73.73%	miR-31 over-expression and epithelial dysplasia synergistically predict OPMD progression.	miR-31 and epithelial dysplasia were independent factors for OPMD progression.	-	[28]
salivary from 36 healthy participants and 36 OPMD patients	0.82	69%	66%	-	-	-	[57]
Serum samples and tissue specimens from 218 patients with OSCC and 90 controls	0.920	0.842	0.810	Patients having elevated serum miR-626 and miR-5100 had significantly decreased DFS and OS. The two-miRNA signature exhibited greater prognostic performance than one-single-miRNA.	The expression of the two risk miRNAs (miR-626 and miR-5100) was inversely related to DFS. Significant associations between DFS and grade, serum miRNA signature, and TNM stage were detected.	The association between this two-miRNA signature and survival was independent of other clinicopathologic variables.	[58]
11 paired match tumor tissues and adjacent tissues, sera from 82 oral cancer patients and 53 normal subjects	0.776 for miR-31-5p	76.8%	73.6%	-	-	-	[55]
51 Samples of HNSCC cancer tissue and adjacent non-cancerous tissue	0.719	93%	61%	Patients with increased expression of miR-15b-5p have a significantly longer locoregional relapse-free survival. The predictive value of miR-15b-5p was independent of other clinicopathological factors, including the stage or the p16 status.	Forty-one out of these forty-six miRNAs were significantly associated with LRC; eleven miRNAs decreased and thirty miRNAs increased the risk of LRC in HNSCC patients.	miR-15b-5p is significantly associated with LRC.	[68]
60 fresh-frozen tissue of tongue carcinomas	0.7 for miR-183	86.2% for miR-183	48.4% for miR-183	Patients with miR-183 up-regulation had shorter overall survival. miR-21 over-expression had a tendency towards poorer survival.	Patients with high miR-183 expression have a 5.6 times higher overall mortality rate, and a tendency towards recurrence.	The recurrences were independent adverse prognostic factors, while miR-183 over-expression lost its significance.	[36]
salivary samples from 150 HNSCC patients and 80 healthy subjects	0.77 for miR-let-7a-5p, 0.78 for miR-3928	-	-	-	-	-	[141]

## 6 Discussion

HNSCC is among common human malignancies and affects more than 600000 individuals yearly [148]. Chemotherapy, radiotherapy and surgical resection are therapeutic modalities that have improved survival of patients; yet, less than half of all patients are rescued [149]. Thus, there is an urgent need for identification of cancer at early stages. Mutations in TP53, proliferation pathways (RAS/PI3K/mTOR pathway, PIK3CA, HRAS), cell cycle regulating genes, Notch pathway, cell communication and death pathways have been identified in HNSCC [150]. Notably, several targets within these pathways are regulated by miRNAs as well. Thus, aberrant expression of miRNAs is an alternative route for development of HNSCC. Expression profiling has revealed dysregulated expression of several miRNAs in HNSCC in association with clinical determinants of cancer behavior; therefore, miRNAs have prominent roles in the pathogenesis of HNSCC. Some preliminary studies have reported correlations between expression profile of miRNAs and site of the HNSCC tumor [9], which might indicate a site-specific role for these miRNAs. Moreover, miRNAs profile is important in the recognition of minimal residual disease in HNSCC [151]. Consistent with this speculation, altered expression of miRNAs in the tumor-adjacent mucosa has been correlated with the risk of HNSCC recurrence [11]. Decreased expression levels of a number of miRNAs such as HNnov-miR-2, HNnov-miR-30, and HNnov-miR-125 have been associated with the presence of HPV infection [23]. Future studies are required to find a putative panel of miRNAs which specifically correlate with HPV status. Several panels of miRNAs have been suggested as diagnostic panels for HNSCC. Yet, diagnostic power of none of them has been verified in large scales. A recent meta-analysis have shown consistent results about aberrant expression of 22 miRNAs including miR-9 and miR-483-5p in HNSCC. Notably, up-regulation of miR-9 and downregulation of miR-483-5p have been associated with poor survival of patients [152]. Other miRNAs such as mIR-191, miR-21, miR-375, miR-31, miR-626, miR-5100, miR-183 and miR-15b-5p are also involved in determination of patients’ prognosis. Levels of miRNAs in the saliva samples might be used for detection of oral SCC both at the time of cancer diagnosis and after resection of the primary tumor [21]. In a retrospective study, Ahmad et al. have shown that miR-15b-5p is differentially expressed between patients with short and long time of locoregional control in a way that patients with higher levels of this miRNA had a remarkably longer locoregional relapse-free survival [68]. Further prospective studies are needed to verify whether expression level of this miRNAs might be employed for individualized treatment decisions. Moreover, plasma levels of a panel of miRNAs including miR-142-3p, miR-186-5p, miR-195-5p, miR-374b-5p and miR-574-3p has been regarded as an HPV-independent prognostic panel for HNSCC patients who were treated with combined radiochemotherapy [16]. miRNAs might also modulate response of cancer cells to chemotherapeutic agents, radiotherapy or even targeted therapies. Besides, preliminary results from cell line studies indicated that suppression and forced expression of a number of miRNAs could influence cancer cells behavior. Thus, miRNAs have been regarded as therapeutic targets. Delivery of certain pre-miRNAs or siRNAs using nanoparticles has been promising [153,154]. Future studies should assess the efficacy of these methods in combination with routine therapeutic options such as chemotherapy. Taken together, miRNAs signature has practical implications in the diagnosis, staging, and management of HNSCC [155,156]. The most important usefulness of miRNAs in HNSCC is their application as diagnostic markers for primary diagnosis of this type of cancer and patients' follow-up. Altered expression levels of miRNAs might reflect tumor recurrence after initial response to the therapeutic options. The stability of miRNAs in the serum samples potentiates these transcripts as suitable tools in non-invasive methods of cancer diagnosis. The therapeutic usefulness of miRNAs have been evaluated in xenograft models of HNSCC, yet clinicasl studies are missing in this regard. Future studies should focus on identification of modalities to restore function of tumor suppressor miRNAs and abolish effects of onco-miRs in animal models as well as clinical settings.

## Declarations

### Author contribution statement

All authors listed have significantly contributed to the development and the writing of this article.

### Funding statement

This work was supported by 10.13039/501100005851Shahid Beheshti University of Medical Sciences.

### Declaration of interests statement

The authors declare no conflict of interest.

### Additional information

No additional information is available for this paper.
